# Utilization of HEPES for Enhancing Protein Transfection into Mammalian Cells

**DOI:** 10.1016/j.omtm.2018.12.005

**Published:** 2018-12-20

**Authors:** Shun-Hua Chen, Angel Chao, Chia-Lung Tsai, Shih-Che Sue, Chiao-Yun Lin, Yi-Zong Lee, Yi-Lin Hung, An-Shine Chao, Ann-Joy Cheng, Hsin-Shih Wang, Tzu-Hao Wang

**Affiliations:** 1 Graduate Institute of Biomedical Sciences, Chang Gung University, Taoyuan, Taiwan; 2 Department of Obstetrics and Gynecology, Chang Gung Memorial Hospital Linkou Medical Center and Chang Gung University, Taoyuan, Taiwan; 3 Gynecologic Cancer Research Center, Chang Gung Memorial Hospital Linkou Medical Center, Taoyuan, Taiwan; 4 Genomic Medicine Research Core Laboratory, Chang Gung Memorial Hospital Linkou Medical Center, Taoyuan, Taiwan; 5 Institute of Bioinformatics and Structural Biology, National Tsing Hua University, Hsinchu, Taiwan; 6 Department of Medical Biotechnology and Laboratory Science, College of Medicine, Chang Gung University, Taoyuan, Taiwan

**Keywords:** HEPES, protein transfection, intracellularly targeting

## Abstract

The delivery of active proteins into cells (protein transfection) for biological purposes offers considerable potential for clinical applications. Herein we demonstrate that, with a readily available, inexpensive organic agent, the 4-(2-hydroxyethyl)-1-piperazineethanesulfonic acid (HEPES) method can be used for simple and efficient protein transfection. By mixing proteins with a pure HEPES solution before they are applied to live cells, proteins with various molecular weights (including antibodies, recombinant proteins, and peptides) were successfully delivered into the cytoplasm of different cell types. The protein transfection efficiency of the HEPES method was not inferior to that of commercially available systems that are both more expensive and time consuming. Studies using endocytotic inhibitors and endosomal markers have revealed that cells internalize HEPES-protein mixtures through endocytosis. Results that HEPES-protein mixtures exhibited a low diffusion coefficient suggest that HEPES might neutralize the charges of proteins and, thus, facilitate their cellular internalization. Upon internalization, the cytosolic antibodies caused the degradation of targeted proteins in TRIM21-expressing cells. In summary, the HEPES method is efficient for protein transfection and has potential for myriad clinical applications.

## Introduction

In this decade, we have witnessed the increased use of antibodies, recombinant proteins, and peptides in preclinical and clinical studies.^1^, ^2^, ^3^, ^4^, ^5^ As a powerful tool in cell biology, the transfection of functional proteins acts as a chemical cue for biological purposes, and it offers considerable potential for clinical applications. Protein transfection has also been useful for obtaining information on protein-protein interactions and protein trafficking within cells. Moreover, it is an essential prerequisite to antibody therapy and vaccinations against intracellular proteins. Therefore, the attention of the biomedical community has been focused on seeking efficient and nontoxic methods of protein transfection.^1^, ^6^

Current delivery systems for the transfer of proteins through the cell membrane to the cytosol include physical (e.g., microinjection and electroporation) and chemical (e.g., macromolecular carriers) systems.^7^, ^8^, ^9^, ^10^, ^11^ In general, the efficiency of delivery into cells depends on the numerous biochemical properties of the proteins (including structure, composition, size, molecular weight, and charge).^12^, ^13^ Commercially available protein transfection chemical reagents include (1) lipid-based, (2) cationic-polymer-based, and (3) cell-penetrating peptide-based formulations.^7^, ^14^Unfortunately, access to such transfection reagents as PULSin,^15^ ProteoJuice,^16^ Xfect,^17^ and BioPorter^18^ is limited by their high cost, which hampers the scalability of the process.

For various proteins, endocytosis is crucial to their cytoplasmic delivery.^19^, ^20^ The mechanisms involved in endocytosis can be classified into groups (i.e., macropinocytosis and caveolin-mediated, clathrin-mediated, clathrin-independent, and caveolin-independent endocytosis), according to the protein machinery employed.^19^, ^21^, ^22^ The treatment of cells with endocytosis inhibitors capable of blocking a specific endocytotic pathway is useful for determining which and how many uptake pathways are utilized by a specific particle or complex.^17^, ^20^, ^23^, ^24^, ^25^ In addition to endocytotic uptake, cytosolic delivery is modulated by subsequent endosomal escape, the promotion of which can also improve biological therapy by ensuring cytosolic delivery.^12^

Commonly used in several biochemical reactions, 4-(2-hydroxyethyl)-1-piperazineethanesulfonic acid (HEPES) is employed as a zwitterionic buffering agent in cell culture media. Indeed, HEPES-buffered solutions are used to introduce plasmid DNA to monolayer cell cultures during calcium phosphate transfection.^26^ The application of HEPES for cellular functions could be as previously described. Possibly as the result of the zwitterionic property of HEPES, HEPES buffer resulted in less diffusion of PrP106-126 peptide in mica-supported lipid bilayers.^27^ Additionally, HEPES repressed the contractility of arterial smooth muscle, and it may promote alkaline-initiated contraction in non-mammalian vessels, indicating the functional effects of HEPES in cells.^28^, ^29^ However, it is still unknown whether it was necessarily the HEPES in these studies that promoted protein delivery in cells.

In this study, we demonstrated for the first time that the HEPES method can be used for efficient protein transfection. After being combined with a pure HEPES solution, proteins with various molecular weights, including antibodies, recombinant proteins, and small peptides, were successfully delivered into the cytosol of various cultured cell lines without notable cytotoxicity. Stress-induced phosphoprotein 1 (STIP1) is upregulated in certain cancers, and suppressing the function of STIP1 inhibits tumor progression.^30^, ^31^ The HEPES method successfully delivered STIP1 antibodies to cancer cells, leading to protein degradation of STIP1. Overall, our data demonstrate that HEPES is a readily available, simple, and efficient reagent for promoting protein transfection and intracellular targeting.

## Results

### Optimization of HEPES Concentration, Incubation Time, Protein-to-HEPES Ratio, and Culture Media for Protein Transfection

To ascertain the optimal HEPES concentration for promoting protein transfection, we mixed purified v5-tagged recombinant human STIP1 (rhSTIP1) protein with pure HEPES solutions of 5- or 20-mM concentration for 15 min. For the 0-mM HEPES control, the protein was mixed with Opti-MEM (containing 20 mM HEPES as a buffering agent) for 15 min. Transfection mixtures were then added to MDAH2774 cells and cultured for 4 or 24 h. The efficiency of protein transfection was assessed using flow cytometry with anti-v5 antibodies.

**Figure 1:**
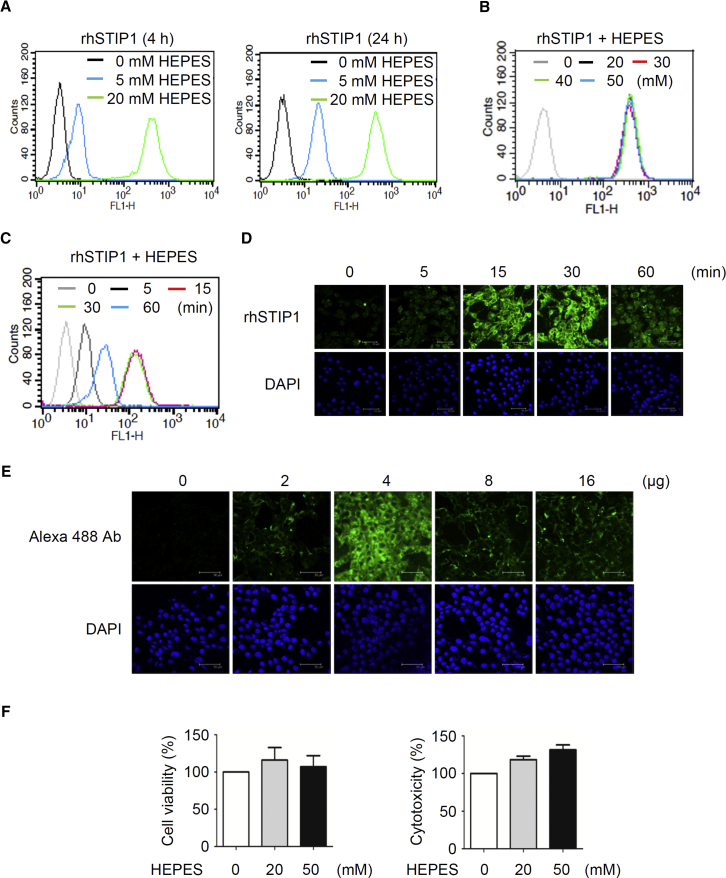
Optimization of the Experimental Conditions for HEPES-Mediated Protein Transfection (A) v5-tagged rhSTIP1 proteins were mixed with either a control Opti-MEM or pure HEPES solution (concentration: 5 or 20 mM) for 15 min before being applied to MDAH2774 cells cultured in Opti-MEM. Afterward, the cells were cultured for 4 or 24 h and assayed using flow cytometry with anti-v5 antibodies. (B) rhSTIP1 proteins were mixed with the control Opti-MEM or a pure HEPES solution (concentration: 20–50 mM) for 15 min before being applied to MDAH2774 cells cultured in Opti-MEM. The cells were subsequently cultured for 24 h and assayed using flow cytometry with anti-v5 antibodies. (C and D) rhSTIP1 proteins were mixed with the control Opti-MEM or a pure HEPES solution (concentration: 20–50 mM) for 15 min before being applied to MDAH2774 cells cultured in Opti-MEM. The cells were subsequently cultured for 24 h and assayed using flow cytometry (C) or immunofluorescent staining (D) with anti-v5 antibodies. (E) MDAH2774 cells were subjected to HEPES-induced (20 mM) protein transfection of Alexa Fluor 488-conjugated anti-mouse IgG antibodies (0–16 μg) in a 6-well plate. After transfection, cells were incubated for 24 h and analyzed using a confocal fluorescent microscope. (F) MDAH2774 cells were cultured in Opti-MEM, to which additional HEPES (additional concentrations: 0–50 mM) was added for 24 h. Cell viability and cytotoxicity were analyzed using MTT and LDH assays, respectively. Error bars indicate the SEM (n = 3). rh, recombinant human; Ab, antibody.

After both 4 and 24 h of incubation, the cells exposed to the protein-20-mM HEPES mixture contained more proteins than did the cells exposed to the mixture containing 5 mM HEPES (Figure 1A). Further increases in HEPES concentration (30, 40, and 50 mM) did not result in greater protein transfection (Figure 1B). A longer incubation time (24 h) slightly improved the efficiency of transfection for a 5-mM HEPES concentration; however, the amount of proteins delivered intracellularly using this concentration was lower than that using 20 mM HEPES (Figure 1A). These results demonstrated that protein transfection was most efficient when the proteins were mixed with a pure 20-mM HEPES solution. Although Opti-MEM also contains 20 mM HEPES as a buffering agent, mixing proteins with Opti-MEM (0 mM HEPES) did not promote protein transfection (Figures 1A and 1B).

We subsequently investigated the effects of reaction time and protein-to-HEPES ratio on transfection into MDAH2774 cells. Purified rhSTIP1 proteins were mixed with 20 mM HEPES at different points in time (between 0 and 60 min). The efficiency of protein transfection at each point in time was compared using immunofluorescent confocal microscopy. The optimal reaction time for the rhSTIP1 protein-HEPES mixture was between 15 and 30 min (Figures 1C and 1D). Furthermore, we tested the efficiency of the 20-mM concentration of HEPES to deliver different amounts of Alexa Fluor 488 anti-mouse immunoglobulin G (IgG) (0, 2, 4, 8, or 16 μg) to MDAH2774 cells in a 6-well plate platform. The most successful intracellular transfection was observed with proteins weighing 4 μg over a reaction time of 24 h (Figure 1E). Surprisingly, higher antibody concentrations (8 and 16 μg) limited the transfection efficiency. Altogether, these results demonstrated the imperativeness of the protein-to-HEPES (mass-to-volume) ratio in the transfection mixture, which should be approximately 1 μg to 50 μL.

To examine whether the addition of HEPES affected cell viability and cytotoxicity in the HEPES method, MDAH2774 cells were cultured in Opti-MEM with additional protein-HEPES mixtures (the concentrations of HEPES were 0, 20, and 50 mM) for 24 h (Figure 1F). The additional HEPES had no effect on cell viability and cytotoxicity, suggesting the low cytotoxicity of the HEPES method. Therefore, various concentrations of 30 and 50 mM HEPES in different cells were used without concerns.

In summary, mixing proteins with a pure 20-mM HEPES solution for 15 min at the ratio of 1 μg to 50 μL is an excellent starting point for the optimization of protein transfection for any type of cell. The increased concentration of HEPES derived from the protein-HEPES mixture in the HEPES method did not appear to have any adverse effect on cell viability.

### The HEPES Method Transfects Antibodies, Recombinant Proteins, and Small Peptides into Cells

**Figure 2:**
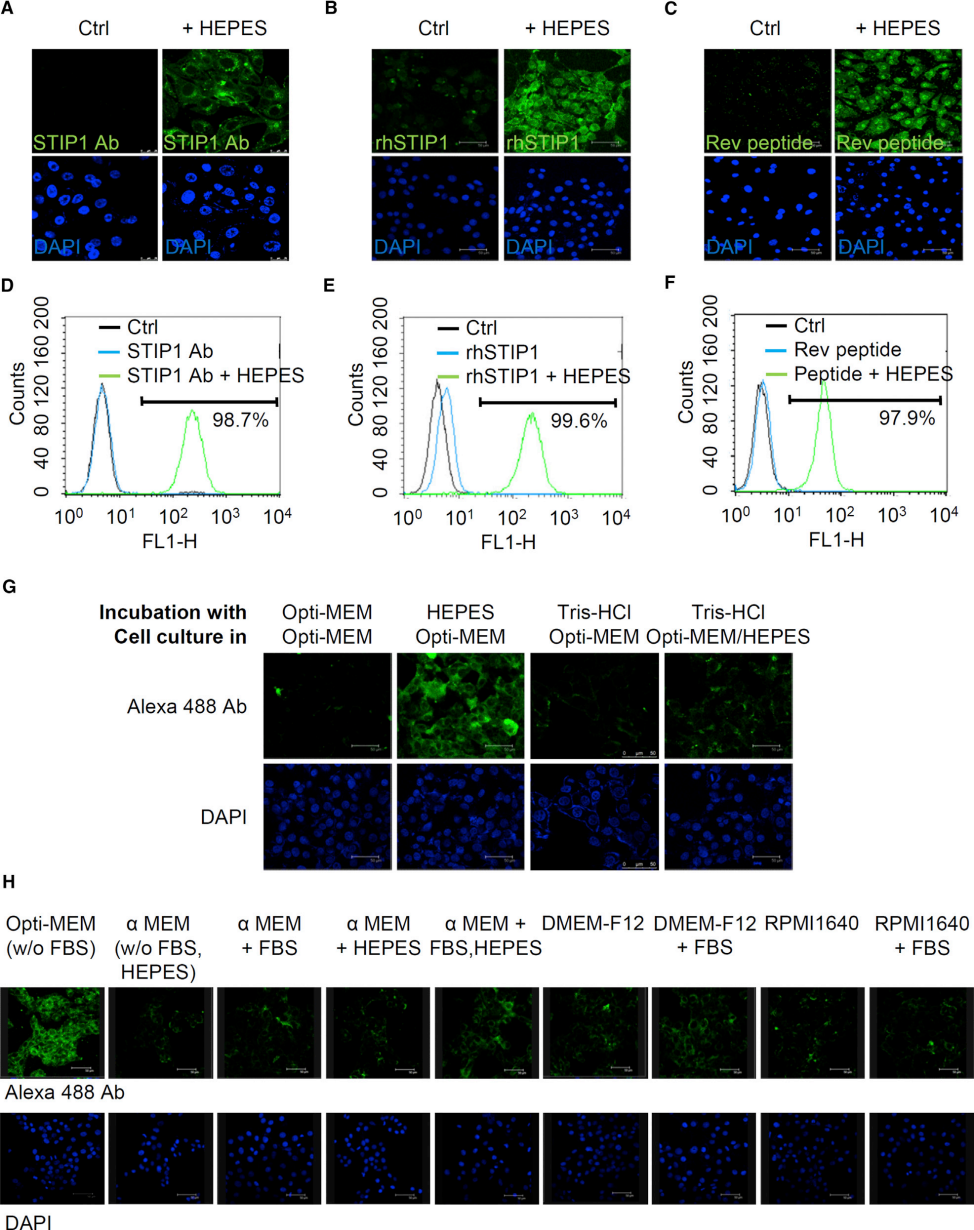
The HEPES Method Was Successful for the Transfection of Proteins of Various Molecular Weights, Including Antibodies, Recombinant Proteins, and Small Peptides (A) Anti-STIP1 antibodies were mixed with Opti-MEM (control [Ctrl]) or a pure 20-mM HEPES solution for 15 min before being applied to ARK2 cells cultured in Opti-MEM. After 24 h of incubation, the cells were stained with Alexa Fluor 488-conjugated antibodies and analyzed using a confocal fluorescent microscope. (B) Purified rhSTIP1 proteins were mixed with Opti-MEM (Ctrl) or a pure 20-mM HEPES solution for 15 min before being applied to MDAH2774 cells cultured in Opti-MEM. After 24 h of incubation, the efficiency of rhSTIP1 protein transfection was determined using immunofluorescent staining with anti-v5 antibodies. (C) FITC-Rev peptides were delivered into MOSEC cells using Opti-MEM (Ctrl) or HEPES (30 mM), as described in The HEPES Method. After 24 h of incubation, the efficiency of FITC-Rev peptide transfection was determined using immunofluorescent staining. The transfection efficiency was also tested by flow cytometry as these results shown in (D) anti-STIP1 antibodies, (E) rhSTIP1 proteins, or (F) FLAG-Rev peptides. These proteins were preincubated with a pure 20-mM HEPES solution for 15 min before being added to MDAH2774 cells cultured in Opti-MEM. After 24 h of incubation, the delivery efficiency was quantified using flow cytometry. (G) Only the preincubation of protein or peptides with the pure HEPES solution promoted protein transfection. Alexa Fluor 488 anti-mouse IgG antibodies were mixed with only Opti-MEM, HEPES (20 mM), or Tris-HCl (20 mM) for 15 min. Antibody and HEPES mixtures were then added to MDAH2774 cells and cultured in Opti-MEM for another 24 h. To determine whether the sole addition of HEPES into Opti-MEM could promote protein transfection, proteins that had not been preincubated with 20 mM HEPES were added to cells that had been cultured in Opti-MEM with an additional 1.3 mM HEPES, which produced the same final HEPES concentration in the culture medium as in the HEPES-incubating experiments. The transfection efficiency was analyzed using a confocal microscope. (H) Opti-MEM is the most suitable culture medium for the HEPES method. Alexa Fluor 488-conjugated anti-mouse IgG antibodies were delivered to MDAH2774 cells using 20 mM HEPES and cultured for another 24 h. After transfection, the cells were incubated in Opti-MEM (containing 20 mM HEPES), α-MEM (either without HEPES or with an additional 20 mM HEPES), DMEM-F12 (containing 15 mM HEPES), or RPMI 1640 (containing 25 mM HEPES) in the presence and absence of 10% FBS; cells were subsequently analyzed using a confocal fluorescent microscope.

Next, we investigated the ability of the HEPES method to transfect proteins of various sizes, such as antibodies, recombinant proteins, and small peptides. Cells assessed using immunofluorescent staining revealed that, in comparison with the process of adding proteins to the control Opti-MEM, mixing proteins with a pure HEPES solution prior to testing (as described in The HEPES Method) resulted in greater intracellular transfection of anti-STIP1 antibodies and rhSTIP1 (Figures 2A and 2B). We subsequently employed flow cytometry to quantify the efficiency of the HEPES-induced transfection of anti-STIP1 antibodies into cells; 98.7% and 99.6% of the cells presented a positive signal for the anti-STIP1 antibody and rhSTIP1, respectively (Figures 2D and 2E). We also compared the efficiency of the HEPES method in promoting protein transfection with the currently commercially available systems. Specifically, PULSin, ProteoJuice, Pro-Ject, Xfect, and BioPORTER QuikEase^17^, ^23^ were compared with the HEPES method. The results indicated that the efficiency of the HEPES method in promoting protein transfection was not inferior to those commercial systems in MDAH2774 cells (Figure S1).

After the successful transfection of IgG antibodies (molecular weight: approximately 150 kDa) and rhSTIP1 proteins (molecular weight: 62.6 kDa) into human endometrial cancer ARK2 cells (Figure 2A) and human ovarian cancer MDAH2774 cells (Figures 2B, 2D and 2E), we tested whether fluorescein isothiocyanate (FITC)-conjugated or FLAG-tagged Rev peptides (molecular weight <5 kDa) could also be delivered into mouse ovarian surface epithelial cancer (MOSEC) (Figure 2C) or MDAH2774 cells (Figure 2F). The Rev peptide is a 13-amino-acid fragment of the HIV, exhibiting high affinity with nucleolar phosphoprotein B23. FLAG-tagged, FITC-conjugated Rev peptides cannot spontaneously enter MOSEC cells, but the peptides were transfected into MOSEC nuclei when 30 mM HEPES was employed in the HEPES method (Figure 2C). Detection of the FLAG-tagged Rev peptides was also performed using flow cytometry with anti-FLAG antibodies in MDAH2774 cells (Figure 2F).

### Preincubation of Protein or Peptides with the Pure HEPES Solution Promoted Protein Transfection

Owing to the zwitterionic properties (the presence of both positive and negative charges) of HEPES, we hypothesized that HEPES might neutralize protein charges for promoting protein transfection. To test this hypothesis, Alexa Fluor 488 antibodies were mixed with 20 mM HEPES or Tris-HCl, and the mixture was subsequently used to treat MDAH2774 cells. The results indicated that HEPES, but not Tris-HCl, promoted antibody transfection into the cells (Figure 2G), suggesting that the interaction between HEPES and proteins is critical for intracellular transfection.

### Opti-MEM Is the Most Suitable Culture Medium for the HEPES Method

Opti-MEM is commonly used to preserve cell life under reduced-serum conditions during DNA transfection. In addition to evaluating the ability of Opti-MEM (containing 20 mM HEPES), we investigated the abilities of α-MEM (containing no HEPES), DMEM-F12 (containing 15 mM HEPES), and RPMI 1640 (containing 25 mM HEPES) to promote protein transfection. Transfected proteins were detected only in the cells that had been cultured in Opti-MEM (Figure 2H). The presence or absence of fetal bovine serum (FBS; 10%) or additional HEPES (20 mM) in the three other media did not improve the transfection efficiency. Owing to its high efficiency and low cytotoxicity, Opti-MEM should be considered the ideal culture medium for HEPES-based protein transfection.

### Applicability of HEPES-Mediated Cytoplasmic Protein Delivery in Multiple Cell Lines

**Figure 3:**
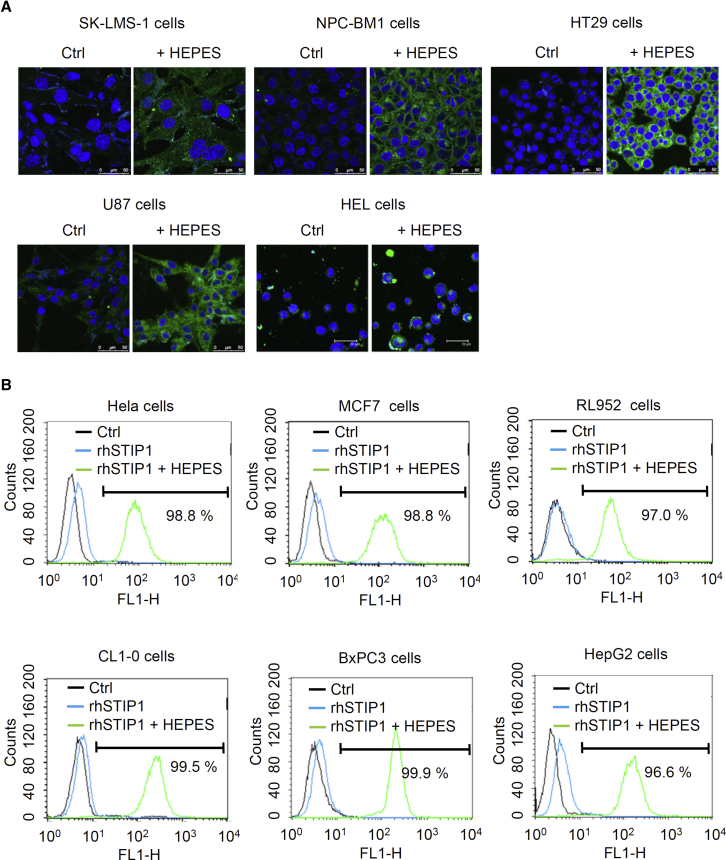
The HEPES Method Was Successful for the Transfection of Proteins into a Variety of Cell Lines (A) Alexa Fluor 488-conjugated anti-mouse IgG antibodies were mixed for 15 min with the control Opti-MEM or various concentrations (shown in parentheses) of pure HEPES solutions according to cell type: SK-LMS-1 (30 mM), NPC-BM1 (20 mM), HT29 (30 mM), U87 (50 mM), and HEL (30 mM). The protein-HEPES mixtures were then added to different cultured cells. After 24 h of incubation, the cells were fixed with 4% paraformaldehyde and analyzed through fluorescent microscopy. (B) Flow cytometry revealed that HEPES successfully promoted protein transfection in a variety of cancer cell lines. We transfected rhSTIP1 proteins using Opti-MEM or different concentrations of pure HEPES solutions in HeLa (30 mM), MCF7 (20 mM), RL952 (20 mM), CL1-0 (20 mM), BxPC3 (30 mM), and HepG2 (20 mM) cells. After 24 h of incubation, the transfection efficiency was determined using flow cytometry.

We investigated whether Alexa Fluor 488 antibodies could be delivered using different concentrations of HEPES in various cultured cell lines, including SK-LMS-1 cells (sarcoma), NPC-BM1 cells (nasopharyngeal carcinoma), HT29 cells (colon cancer), U87 cells (brain tumor), and HEL cells (leukemia). The results of confocal microscopy revealed that HEPES could promote protein transfection in various cancer cells (Figure 3A). Using the HEPES method, we also tested the transfection of rhSTIP1 proteins in other cell lines. The results of flow cytometry (Figure 3B) revealed that HEPES successfully promoted protein transfection in >90% of the tested cancer cells, including HeLa cells (cervical cancer), MCF7 cells (breast cancer), RL952 cells (endometrial cancer), CL1-0 cells (lung cancer), BxPC3 cells (pancreatic cancer), and HepG2 cells (liver cancer), suggesting that the HEPES method works efficiently for protein transfection in most cultured cells.

**Figure 4:**
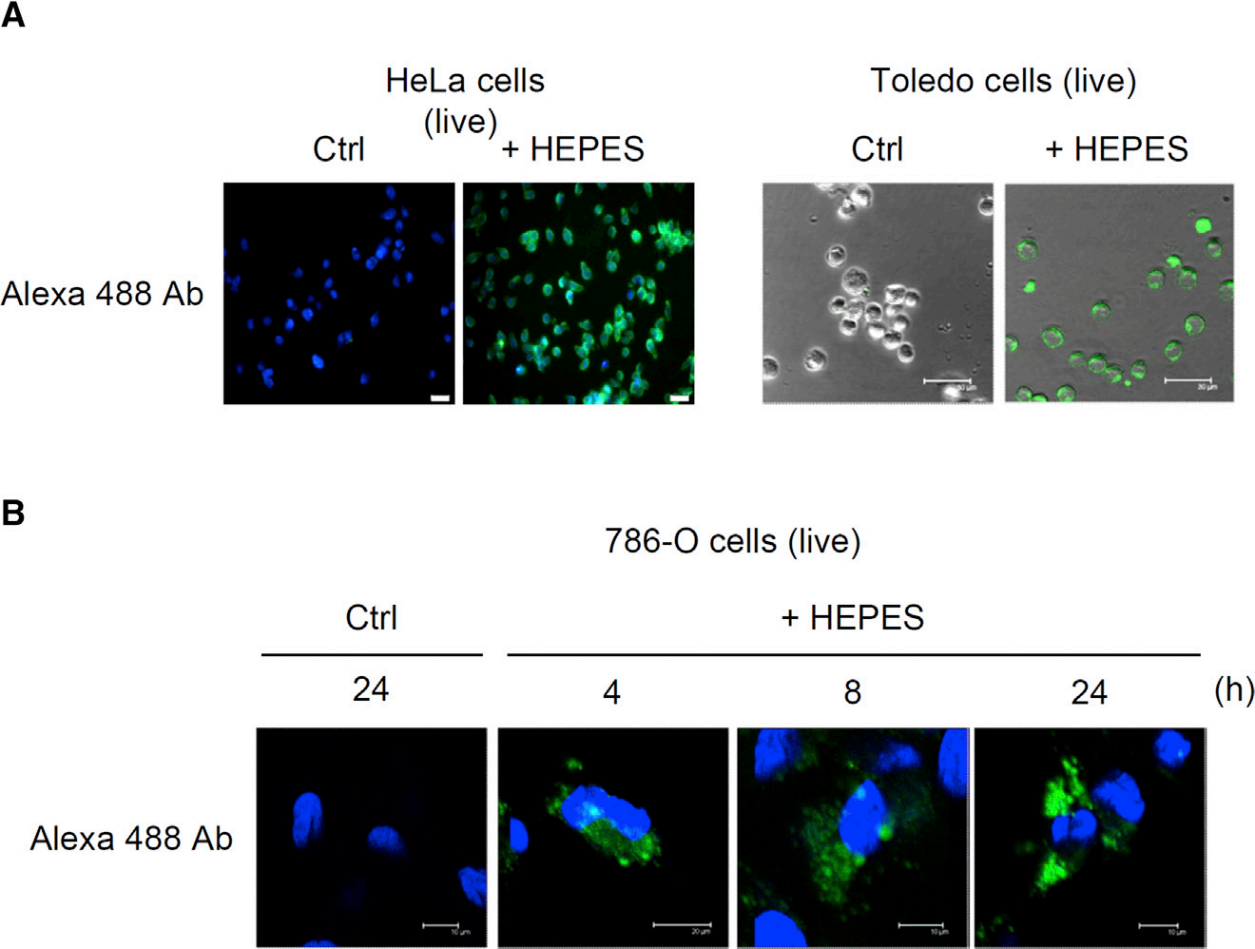
The HEPES Method Was Successful for the Transfection of Proteins into a Variety of Live Cell Lines Alexa Fluor 488-conjugated anti-mouse IgG antibodies were mixed for 15 min with the control Opti-MEM or various concentrations (shown in parentheses) of pure HEPES solutions according to cell type: HeLa (30 mM), Toledo (50 mM), and 786-O (30 mM) cells. After 24 h (A, HeLa and Toledo cells) or 4–24 h (B, 786-O cells) of incubation, the live cells were examined without fixation using fluorescent microscopy.

**Figure 5:**
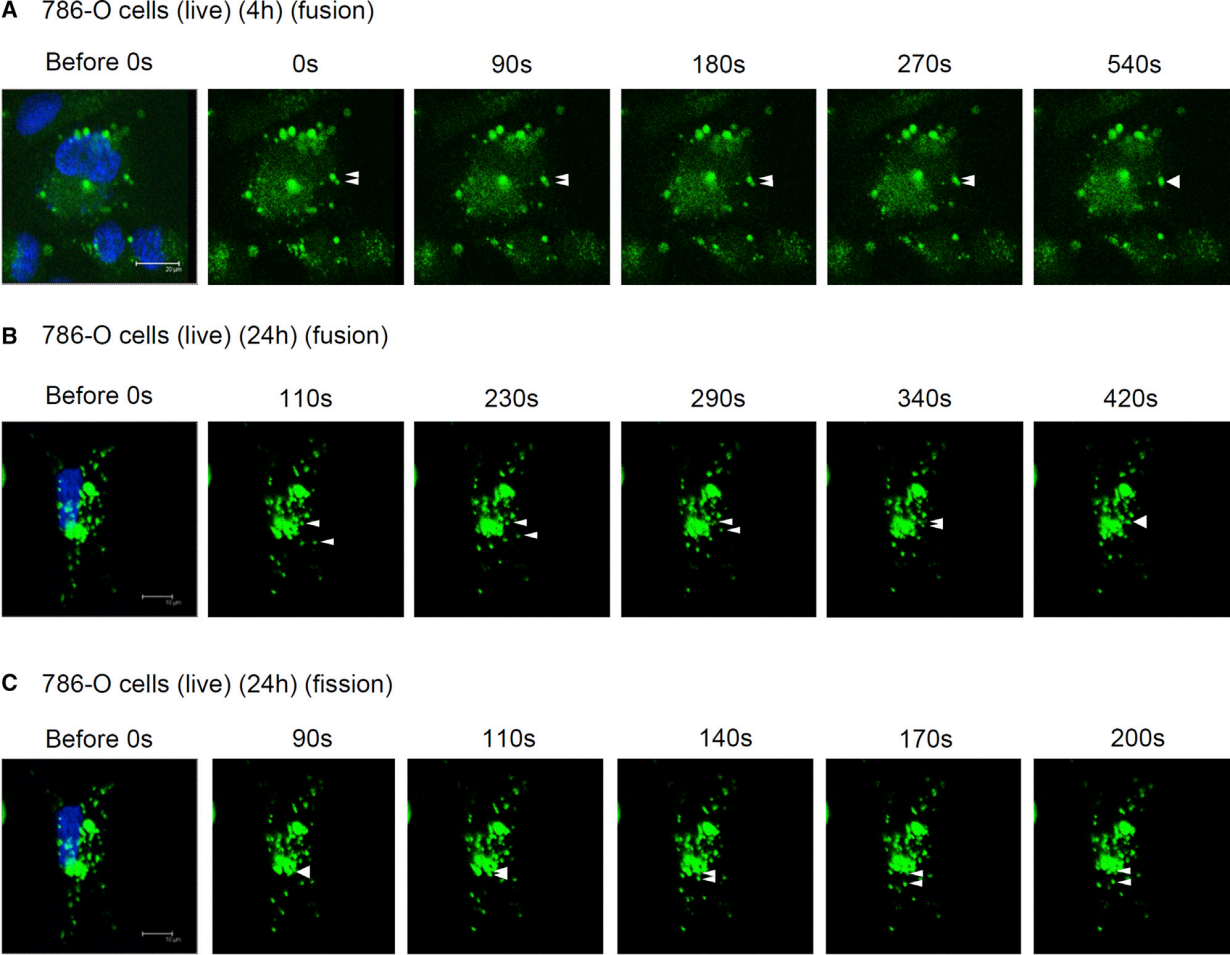
Time-Lapse Fluorescent Microscopy Revealed that HEPES-Promoted Transfection Is an Endocytotic Process in Live 786-O Cells Alexa Fluor 488-conjugated anti-mouse IgG antibodies were incubated with a pure 30-mM HEPES solution for 15 min before being added to 786-O cells. After (A) 4 or (B and C) 24 h of incubation, the live cells were analyzed without fixation using time-lapse fluorescent microscopy. Images were captured every 10 s for periods ranging from 0 to 30 min. White arrows indicate the movement of antibody puncta in live cells. Fusion of the fluorescent puncta was observed in (A) and (B), whereas fission of a fluorescent puncta was observed in (C). For dynamic movements of puncta, please refer to Videos S1, S2, and S3.

To exclude the possibility that the detection of transfected proteins might merely have been the result of an artifact of fixation with paraformaldehyde, we transfected Alexa Fluor 488 antibodies in live cells for 4–24 h before analysis using fluorescent microscopy without fixation. Alexa Fluor 488 antibodies were delivered by prior mixing with 30 and 50 mM HEPES into the cytoplasm of HeLa and Toledo cells (B cell lymphoma), respectively (Figure 4A). Furthermore, live 786-O cells (renal adenocarcinoma) were used, because of their relatively large size, to elucidate the intracellular locations of transfected proteins. During the 4- 24-h examination period, a prior mixing with 30 mM HEPES successfully transferred Alexa Fluor 488 antibodies into the cytoplasm of live 786-O cells, supporting the hypothesis that longer transfection periods (24 h) are preferable to shorter periods (4 or 8 h) (Figure 4B). Alexa Fluor 488 antibodies were also observed in the cytoplasm using time-lapse microscopy (Figure 5; Videos S1, S2, and S3). These results confirmed that the HEPES method is efficient for protein delivery in live cells.

Video S1. 4 h Fusion

Video S2. 24 h Fusion

Video S3. 24 h Fission

### HEPES-Induced Protein Transfection Occurs through Cellular Endocytotic Pathways and Protein-Charge Neutralization

**Figure 6:**
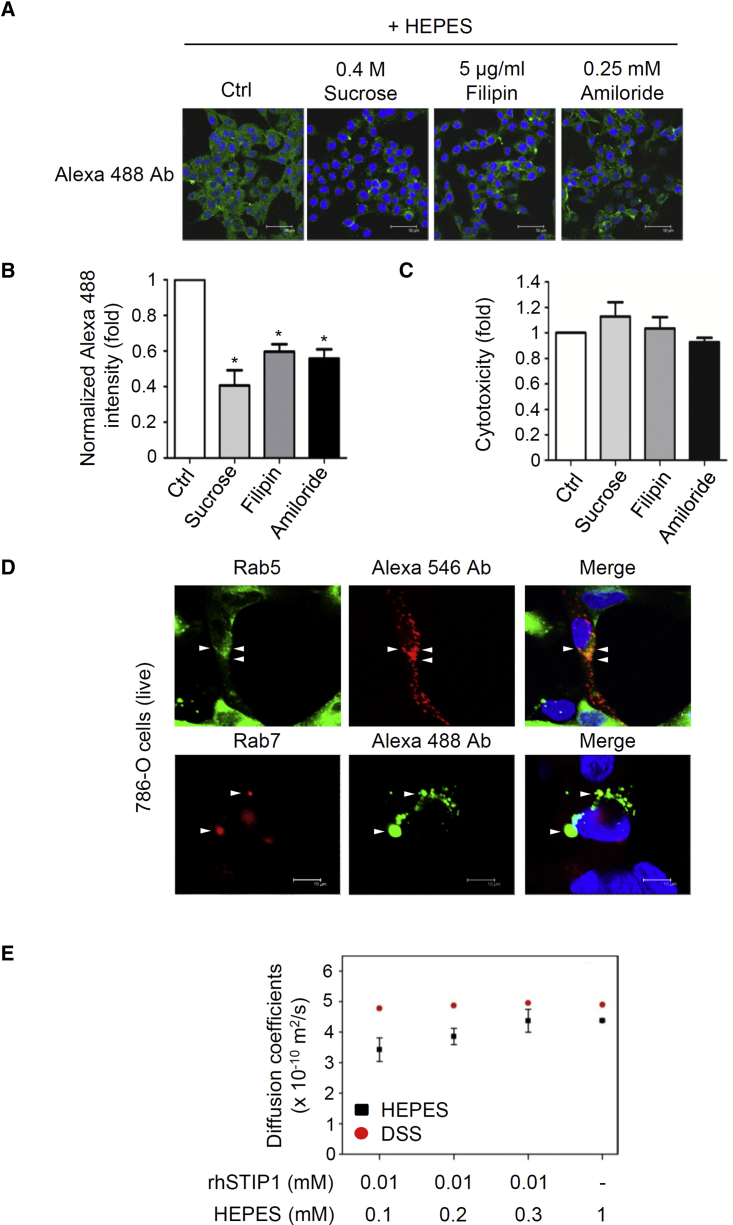
HEPES-Induced Protein Transfection Was Accomplished through Endocytosis and Charge Neutralization (A–C) MDAH2774 cells were pretreated for 30 min with Opti-MEM (Ctrl), 0.4 M sucrose (an inhibitor of clathrin-mediated endocytosis), 5 μg/mL filipin (an inhibitor of caveolae-mediated endocytosis), or 0.25 mM amiloride (an inhibitor of macropinocytosis). Alexa Fluor 488-conjugated antibodies were delivered into cells using 20 mM HEPES, as described in The HEPES Method, followed by a 4-h incubation period. (A) Transfection efficiency was determined using a confocal fluorescent microscope. (B) Alexa Fluor 488 fluorescent intensity signals were quantified using Q-Win software and are expressed as relative ratios. (C) At 4 h of inhibitor treatment, cytotoxicity was analyzed using the LDH assay. Error bars indicate the SEM (n = 3).*p < 0.05. (D) To colocalize transfected proteins with the early endosome, we first used the HEPES method to transfect Alexa Fluor 546-conjugated antibodies into 786-O cells, and then we infected cells with CellLight Early Endosomes-GFP, BacMam 2.0 to express GFP-Rab5. To colocalize transfected proteins with the late endosome, we instead used Alexa Fluor 488-conjugated proteins and CellLight Late Endosomes-RFP, BacMam 2.0 to express RFP-Rab7. After 24 h of incubation, the live 786-O cells were examined using fluorescent microscopy. (E) Self-diffusion coefficients (Ds) were calculated at different molar ratios of STIP1 to HEPES (black squares). The Ds of the NMR internal standard (DSS) obtained under the same experimental conditions served as a reference (red circles). Ctrl, control; Ab, antibody; rh, recombinant human; DSS, dimethyl-silapentane-sulfonic acid.

To further understand the mechanisms through which HEPES promotes protein transfection, we tested the inhibition of cellular uptake of proteins using three endocytic inhibitors: sucrose, a clathrin-mediated endocytotic inhibitor; filipin, a caveolae-mediated endocytotic inhibitor; and amiloride, a macropinocytosis inhibitor.^17^, ^23^ At a concentration that was not cytotoxic, all of the inhibitors blocked the HEPES-mediated transfection of antibodies in MDAH2774 cells (Figures 6A–6C), suggesting that HEPES-induced protein transfection is dependent on endocytotic pathways. To further confirm the critical role of endocytotic pathways, early endosome GFP (GFP-fused Rab5) and late endosome-red fluorescent protein (RFP) (RFP-fused Rab7) were used on live 786-O cells. The results revealed that intracellular Alexa Fluor 488 antibodies existed in both Rab5-positive puncta (endosome) and Rab5-negative puncta (cytoplasm). Similarly, Alexa Fluor 546 antibodies were colocalized with Rab7-positive and Rab7-negative puncta (Figure 6D). Using time-lapse microscopy, both fusion and fission were observed in the Alexa Fluor 488 antibodies in the cytoplasm, demonstrating the dynamic nature of their endocytosis (Figure 5; Videos S1, S2, and S3). Overall, these findings suggest that the HEPES-mediated cytoplasmic delivery of proteins occurs through endocytotic uptake.

We also used nuclear magnetic resonance (NMR) diffusion experiments to assess the diffusion coefficient of HEPES before and after titration into protein solutions. The diffusion coefficient of HEPES was approximately 4.4 × 10^−10^ m^2^/s before the addition of rhSTIP1 proteins (at an rhSTIP1: HEPES molar ratio of 1:10), whereas it was lowered to 3.4 × 10^−10^ m^2^/s after the addition of rhSTIP1 proteins (Figure 6E). The binding of HEPES to rhSTIP1 proteins resulted in a reduced diffusion coefficient, signifying slower diffusion in the solution; however, the internal standard of 4, 4-dimethyl-4-silapentane-1-sulfonic acid (DSS) exhibited no change in its diffusion coefficient, indicating that the solutes did not alter solvent viscosity. As a buffering agent, HEPES is a zwitterionic molecule that brings both positive and negative charges. This particular property affords HEPES the opportunity to accompany an ionic protein and maintain a neutral charge. The result is reflective of a nonspecific interaction between HEPES and the target proteins, suggesting that HEPES may induce intracellular transfection by neutralizing protein charges.

### The Proteins Transfected Using the HEPES Method Are Functional

**Figure 7:**
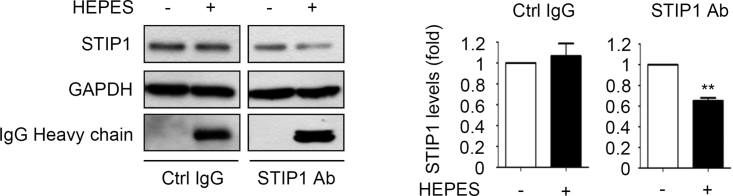
The Efficient HEPES-Mediated Transfection of Antibodies into the Cytosol Mouse IgG_2_ antibodies (Ctrl IgG) or anti-STIP1 antibodies were preincubated in a pure 30-mM HEPES solution for 15 min before being applied to HeLa cells. After 24 h of incubation, STIP1 protein levels were analyzed using western blotting with an anti-STIP1 rabbit antibody. IgG heavy chains were detected with donkey anti-mouse IgG antibodies. Error bars indicate the SEM (n = 3). **p < 0.01, ***p < 0.001.

Several functional assays we reperformed to further determine whether the HEPES-mediated cytosolic delivery of proteins is viable for clinical applications. Cytosolic delivery of antibodies was reported to induce degradation of the targeted proteins through a TRIM21-dependent mechanism in cultured cells.^32^ Mouse IgG antibodies or anti-STIP1 antibodies were transfected using the HEPES method in reportedly TIRM21-positive HeLa cells. After protein transfection, the heavy chains of both types of antibodies were successfully detected using western blotting, but only the anti-STIP1 antibodies triggered the degradation of endogenous STIP1 (Figure 7).

## Discussion

HEPES has previously been used as a buffering reagent in the calcium phosphate transfection method for introducing DNA into mammalian cells. To our knowledge, this study is the first to demonstrate that HEPES is also functional for efficient protein transfection. Although extracellular proteins and cell surface receptors are currently the main targets of protein-based drugs,^1^, ^5^, ^33^, ^34^, ^35^, ^36^, ^37^ the interactions between ligands and cytosol or nuclear receptors may also have therapeutic value. Therefore, our findings may have significant implications for intracellular protein therapy.

Points worth consideration regarding the HEPES method for protein transfection are as follows. First, HEPES incubation time should be adjusted according to the cell type and size of the protein to be delivered. As shown in Figures 1C and 1D, pure HEPES solutions should ideally be incubated with proteins for 15–30 min. Second, the protein:HEPES ratio is another key parameter. We obtained optimal results with a protein mass-to-HEPES volume (μg:μL) ratio of 1:50. Notably, a protein mass of 4 μg dramatically increased the transfection efficiency in a 6-well plate format (Figure 1E). These results suggest that the protein:HEPES ratio has a greater influence than the protein mass on the efficiency of transfection. Third, we discovered that certain cell lines (e.g., MOSEC, HeLa, BxPC3, SK-LMS-1, 786-O, HT29, and HEL cells) required a higher concentration of HEPES (30 mM) than is commonly used (20 mM) (Figures 2, 3, and 4). Because the required HEPES concentration for a prior mixing with proteins was as high as 50 mM for U87 cells and Toledo cells (Figures 3A and 4A), concentration seems to be a crucial parameter influencing the efficiency of protein transfection in different cells. Finally, we found that using Opti-MEM for cell culturing facilitated HEPES-mediated protein transfection (Figure 2H).

HEPES has been used in plasmid DNA transfection, and one study demonstrated that HEPES weakened van der Waals attraction in lipid membranes, ultimately promoting lipid bilayer softening and enabling closer interaction of polar molecules with the membrane surface.^18^ Lipid bilayers are largely impermeable to charged molecules, and neutral proteins can cross the membrane remarkably quicker than charged molecules can. Because the presence of proteins reduced the diffusion coefficient of HEPES (Figure 6E), we reasoned that HEPES promoted intracellular transfection by reducing protein polarity; however, HEPES was unable to modify protein surface hydrophobicity (data not shown). This observation further strengthened the idea that HEPES promotes protein transfection primarily through the reduction of protein charges.

Proteins are known to use multiple pathways for their endocytotic entry into eukaryotic cells.^19^ Large molecules (200–500 nm) are generally internalized through caveolae-dependent endocytosis or macropinocytosis, whereas small molecules (<200 nm) are usually internalized through clathrin-dependent endocytosis.^38^ The internalization mechanisms of commercially available protein transfection systems (e.g., Pro-DeliverIN, Xfect, and Turbofect) into HeLa cells are reported to potentially vary.^17^ Cell line-dependent variation has been observed in both DNA and protein transfection.^17^, ^23^, ^24^ For example, the uptake of Xfect by Huh-7 occurs through caveolae-mediated endocytosis, whereas its internalization into HeLa cells involves both caveolae-mediated endocytosis and macropinocytosis.^23^The results in the present study of using three endocytic inhibitors suggest that, in the HEPES method, proteins enter cells through macropinocytosis, clathrin-dependent endocytosis, and caveolae-dependent endocytosis (Figures 6A and 6B). Additionally, proteins transfected through the HEPES method were discovered to present with both endosomal and cytoplasmic puncta in live 786-O cells (Figure 6D). The time-lapse dynamics of the fusion and fission of transfected protein signals that were observed in live 786-O cells (Figure 5; Videos S1, S2, and S3) are also typical endocytotic characteristics. Overall, these results indicate that HEPES-promoted cytoplasmic delivery of proteins is mediated by endocytotic processes.

The cytosolic delivery of antibodies in documented cases has been performed through microinjection, electroporation, delivery reagents, and signal peptides. The stability or function of antibodies in cytosol has been used to prove successful protein delivery to cells.^32^, ^39^, ^40^ STIP1^41^ is a co-chaperone that is overexpressed in a number of malignancies.^30^, ^31^, ^42^, ^43^, ^44^, ^45^, ^46^, ^47^, ^48^, ^49^ In the present study, we used targeting STIP1 for an anticancer therapeutic strategy as a demonstration of the efficiency, specificity, and clinical potential of protein transfection through the HEPES method.

A TRIM21-mediated Trim-Away mechanism has been used to explain how a transfected antibody causes protein degradation in corresponding endogenous targets.^32^ In TRIM21-expressing HeLa cells, the HEPES method transfected anti-STIP1 antibodies and decreased endogenous STIP1 protein levels (Figure 7). These results suggest that the HEPES method can efficiently deliver therapeutic proteins to the cytosol of targeted cells for a variety of purposes.

In summary, the results of this study suggest that the HEPES method may be a simple, inexpensive method of efficient protein transfection, with myriad potential clinical applications.

## Materials and Methods

### The HEPES Method: HEPES-Mediated Protein Transfection

**Figure 8:**
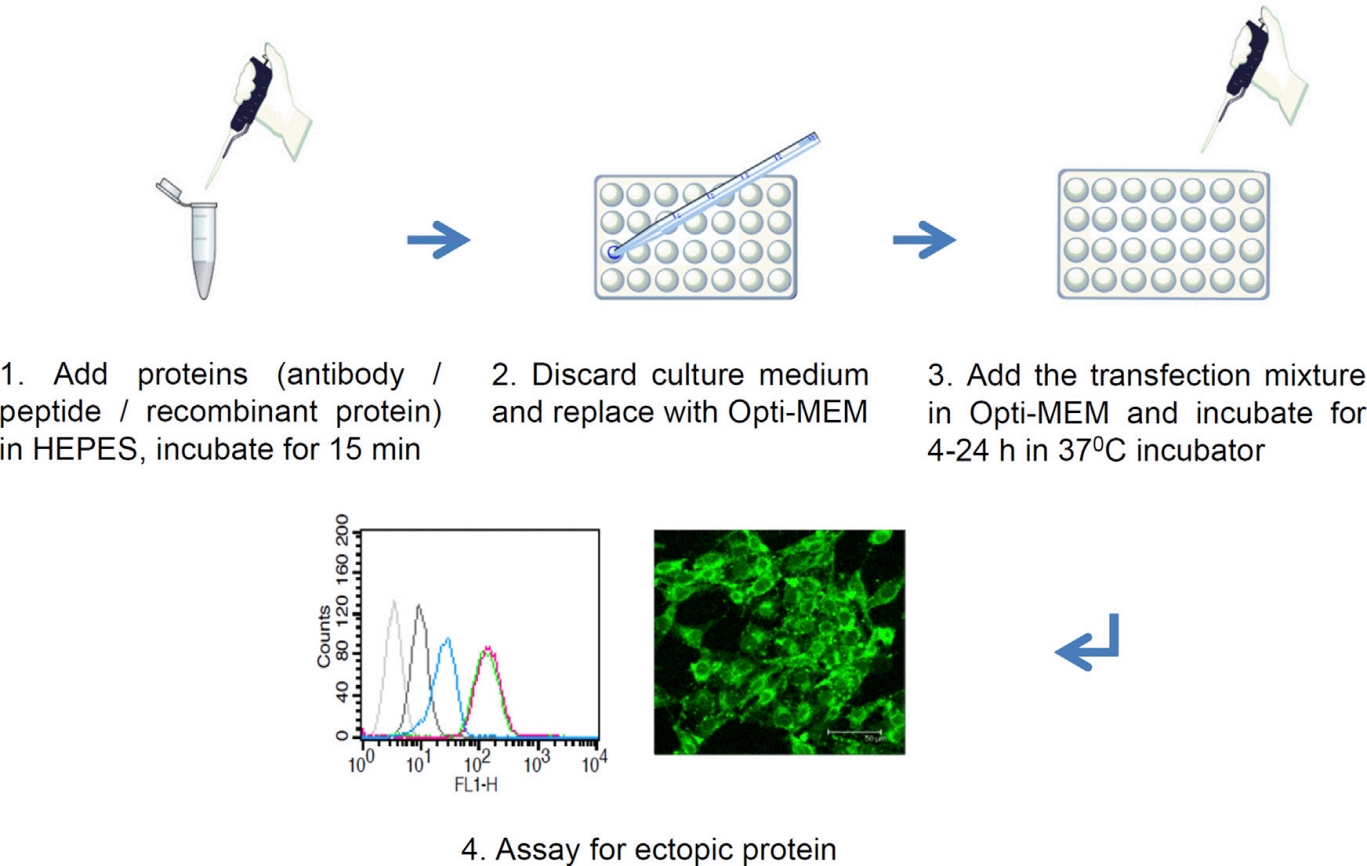
Schematic Representation of the HEPES Method (1) Different proteins (antibodies, recombinant proteins, and small peptides) were mixed with pure HEPES solutions (concentrations 20–50 mM) for 15–30 min. (2) When cells reached 70%–80% confluence, the culture medium was replaced with Opti-MEM. (3) The protein-HEPES mixtures were added to the cultured cells, which were then incubated at 37°C for 4–24 h. (4) The transfected proteins were detectable within the cell between 4 and 24 h thereafter. The presence of transfected proteins could be easily detected using immunofluorescent staining or flow cytometry.

Cells (8 × 10^5^) were plated in 6-cm dishes for 24 h, and their culture media were subsequently replaced by Opti-MEM (5 mL). Different proteins, including antibodies, recombinant proteins, and small peptides (each amount used was 7 μg), were mixed with 400 μL pure 20-mM HEPES (pH 7.4) solution or Opti-MEM (as a negative control) for 15–30 min at room temperature. The transfection mixture was added to cultured cells, which were in Opti-MEM without serum, and incubated at 37°C for 4–24 h. The workflow of the HEPES method is summarized in Figure 8. The amount of individual components in the transfection mixtures can be adjusted according to the size of cultures. For example, when using a 6-well plate, 4 μg protein should be mixed with 200 μL HEPES.

For comparison, the intracellular transfection of proteins was also performed using the following commercial systems, according to the manufacturers’ instructions: Pro-Ject (Pierce Biotechnology, Rockford, IL, USA), Xfect (Clontech Laboratories, Palo Alto, CA, USA), ProteoJuice (Merck Millipore, Darmstadt, Germany), BioPORTER QuikEase (Sigma-Aldrich, St. Louis, MO, USA), and PULSin (Polyplus-transfection, Illkirch, France).

### Cell Cultures

Cells of ovarian cancer (MDAH2774 and SKOV3), mouse ovarian cancer (MOSEC), cervical cancer (HeLa), breast cancer (MCF7), endometrial cancer (RL952), and lung adenocarcinoma (CL1-0) were cultured in DMEM-F12. Cells of pancreatic cancer (BxPC3), endometrial cancer (ARK2), sarcoma cancer (SK-LMS-1), renal cell adenocarcinoma (786-O), nasopharyngeal cancer (NPC-BM1), colorectal adenocarcinoma cancer (HT-29), and leukemia (HEL) were cultured in RPMI 1640. Non-Hodgkin lymphoma cells (Toledo) were cultured in RPMI 1640 containing 1.5 g/L sodium bicarbonate, 4.5 g/L glucose, 10 mM HEPES, and 1.0 mM sodium pyruvate. Hepatocellular carcinoma cells (HepG2) were cultured in DMEM, whereas glioblastoma cells (U87) were maintained in α-MEM. All media were purchased from Invitrogen (Life Technologies, Carlsbad, CA, USA) and contained 10% FBS and 1% penicillin and streptomycin.

Unless otherwise indicated, cell lines were obtained from the American Type Culture Collection (Manassas, VA, USA). Toledo cells originated from the Bioresource Collection and Research Center (Hsinchu, Taiwan). ARK2 cells were kindly provided by Dr. Alessandro D. Santin (Yale University, School of Medicine, New Haven, CT, USA). HEL cells were obtained from Dr. Tsu-Yi Chao (Tri-Service General Hospital, National Defense Medical Center, Taipei, Taiwan), and NPC-BM1 cells were kindly provided by Dr. Chih-Ching Wu (Department of Medical Biotechnology and Laboratory Science, Chang Gung University, Taoyuan, Taiwan). Finally, MOSEC cells were obtained from Dr. Chih-Long Chang (Department of Obstetrics and Gynecology, Mackay Memorial Hospital, Taipei, Taiwan).

### Antibodies, Recombinant Proteins, Peptides, and Chemicals

The antibodies used in this study were Alexa Fluor 546 anti-mouse IgG, Alexa Fluor 488 anti-mouse IgG, v5 (Life Technologies), GAPDH (Santa Cruz Biotechnology, Dallas, TX, USA), FLAG (Sigma-Aldrich, St. Louis, MO, USA), STIP1 mouse (Abnova, Taipei, Taiwan), and STIP1 rabbit (Gentex, Taipei, Taiwan). The procedures used for the production and purification of the v5-tagged rhSTIP1 proteins have been previously described in detail.^31^ The FLAG-Rev peptide consists of a FLAG tag and the Rev peptide, which is a 13-amino-acid fragment of the HIV and has high affinity with the B23 protein.^50^ The commercial sources of the chemicals were as follows. Sucrose and HEPES were from Sigma, filipin was obtained from Santa Cruz Biotechnology, and amiloride hydrochloride was purchased from Toronto Research Chemicals (Toronto, ON, Canada). CellLight Early Endosomes-GFP BacMan 2.0 (C10586) and CellLight Late Endosome-RFP BacMan 2.0 (C10589) were purchased from Thermo Fisher Scientific (Waltham, MA, USA).

### Immunofluorescent Staining

After protein transfection, the cells in culture dishes were washed three times with PBS and fixed with 4% paraformaldehyde for 20 min. After washing with PBS, cells were permeabilized with 0.1% Triton X-100 for 5 min and blocked with 10% BSA for 30 min. In experiments involving Alexa Fluor 488 anti-mouse IgG antibodies, cells were stained with DAPI for 10 min. Regarding the transfection of rhSTIP1 proteins, cells were stained with anti-v5 antibodies and subsequently exposed to Alexa Fluor 488 anti-mouse IgG and DAPI. All fixed cell images were acquired using a confocal microscope with a 63× objective lens and laser excitation and emission of 488/500–535 nm (Leica, Wetzlar, Germany). The fluorescent signals were analyzed and quantified with Q-Win software (Leica).

### Fluorescent Imaging of Live Cells

Cells were cultured in a 30 × 30-mm round glass coverslip. The transfection mixtures (Alexa Fluor 488 anti-mouse IgG and HEPES) were added to Opti-MEM and incubated at 37°C for 4–24 h. After transfection, cells were washed twice with PBS and then retained in Opti-MEM. All live cell images were acquired using a confocal microscope with a 20× objective lens and laser excitation and emission of 488/500–535 nm (Leica, Wetzlar, Germany).

### Flow Cytometry

After 24 h of protein transfection with v5-tagged rhSTIP1 proteins, anti-STIP1 antibodies, or FLAG-Rev peptides, the cells (1 × 10^6^) were washed with PBS, trypsinized, and pelleted. The cells were then mixed with a fixation and permeabilization working solution (eBioscience, San Diego, CA, USA), incubated at 4°C for a specified amount of time (varying from 30 min to 24 h), and then washed twice with 1× permeabilization buffer (eBioscience). To investigate the effectiveness of protein transfection, the cells were stained on ice with either anti-v5 or anti-FLAG antibodies and Alexa Fluor 488 anti-mouse antibodies. Finally, the cells were suspended in 1× PBS and analyzed using flow cytometry (BD Biosciences, San Jose, CA, USA).

### Western Blot Analysis

Cells were lysed in a combination of radioimmunoprecipitation assay buffer (150 mM NaCl, 20 mM Tris-HCl [pH 7.5], 1% Triton X-100, 1% NP-40, 0.1% SDS, and 0.5% deoxycholate) and protease and phosphatase inhibitors (Bionovas, Toronto, ON, Canada). The Bradford assay (Bio-Rad, Hercules, CA, USA) was used to determine the total protein concentration. Each sample (50 μg protein for each) was subjected to SDS-PAGE, and resolved proteins were transferred onto a nitrocellulose membrane. All antibodies and corresponding horseradish peroxidase-conjugated antibodies were acquired from commercial sources. Labeled proteins were visualized using enhanced chemiluminescence (Millipore, Bedford, MA, USA).

### Inhibition of Endocytosis

Cells were pretreated for 30 min with chemicals specifically for inhibiting endocytosis: sucrose (0.4 M) for clathrin-mediated endocytosis, filipin (5 μg/mL) for caveolae-mediated endocytosis, and amiloride (0.25 mM) for macropinocytosis. Control experiments without inhibitor pretreatment were run in parallel. The protein transfection of Alexa Fluor 488 anti-mouse IgG was then elicited through incubation with HEPES (20 mM), followed by an additional 4 h of being cultured with endocytosis inhibitors. Fluorescent signals were acquired using a confocal microscope and were quantified using Q-Win software.

### Viability and Toxicity Assays

Various cell types cultured on 96-well plates were subjected to HEPES-mediated transfection. Cell viability was assessed using the 3-(4,5-dimethylthiazol-2-yl)-2,5-diphenyltetrazolium bromide (MTT) assay; cells subjected to the transfection were incubated for 24 h and then centrifuged at 300 × *g* for 5 min. The supernatant media were discarded, and the attached cells were analyzed using a commercially available MTT assay (Promega, Madison, WI, USA). Cell toxicity was assessed using the lactate dehydrogenase (LDH) assay. To this aim, the cells subjected to the transfection were incubated for 48 h and then centrifuged at 300 × *g* for 5 min. The supernatant media were then collected and analyzed using a commercially available LDH assay (Sigma). The MTT and LDH signals were measured on an ELISA reader (Tecan, San Jose, CA, USA).

### Diffusion NMR

Diffusion NMR experiments were performed at a temperature of 298 K on a Bruker 600-MHz NMR spectrometer (Bruker Daltonics, Bremen, Germany). The NMR sample contained STIP1 at a concentration of 10 μM. HEPES was then titrated into the solution to form different protein:HEPES molar ratios (1:10, 1:20, and 1:30). Diffusion experiments were performed using the Bruker pulse sequence, ledbpgpprwg2s, and the Bruker macro diffusion ordered spectroscopy (DOSY). Self-diffusion coefficients (D_s_) were measured using the Bruker DOSY analysis software for each one-dimensional ^1^H spectrum. HEPES resonances at 3.86, 3.16, and 2.98 ppm were used for the measurements.

### Statistical Analyses

Data are presented as mean ± SEM. Statistical analysis was performed using Prism 5.01 (GraphPad) through Student’s t test, and a significant difference was presumed when p < 0.05.

## Author Contributions

S.-H.C., A.C., and T.-H.W. conceived the study. S.-H.C., C.-L.T., S.-C.S., C.-Y.L., Y.-Z.L., and Y.-L.H. performed the experiments. S.-H.C., S.-C.S., A.-J.C., and T.-H.W. analyzed the results. S.-H.C., A.C., A.-S.C., S.-C.S., H.-S.W., and T.-H.W. wrote the manuscript. All authors read and approved the final manuscript.

## Conflicts of Interest

S.-H.C., A.C., and T.-H.W. filed patent applications for the discovery of the HEPES method for protein transfection.

## Supplemental Information

Document S1. Figure S1

Document S2. Article plus Supplemental Information
