# Supplementary material for: Utilization of HEPES for Enhancing Protein Transfection into Mammalian Cells
Source: Mol Ther Methods Clin Dev. 2018 Dec 20;13:99–111. doi: 10.1016/j.omtm.2018.12.005 (PMC6357789; doi:10.1016/j.omtm.2018.12.005)
Supplement: Document S1. Figure S1 [file mmc1.pdf]

OMTM, Volume *13*

Supplemental Information

Utilization of HEPES for Enhancing Protein Transfection into Mammalian Cells

Shun-Hua Chen, Angel Chao, Chia-Lung Tsai, Shih-Che Sue, Chiao-Yun Lin, Yi-Zong Lee, Yi-Lin Hung, An-Shine Chao, Ann-Joy Cheng, Hsin-Shih Wang, and Tzu-Hao Wang

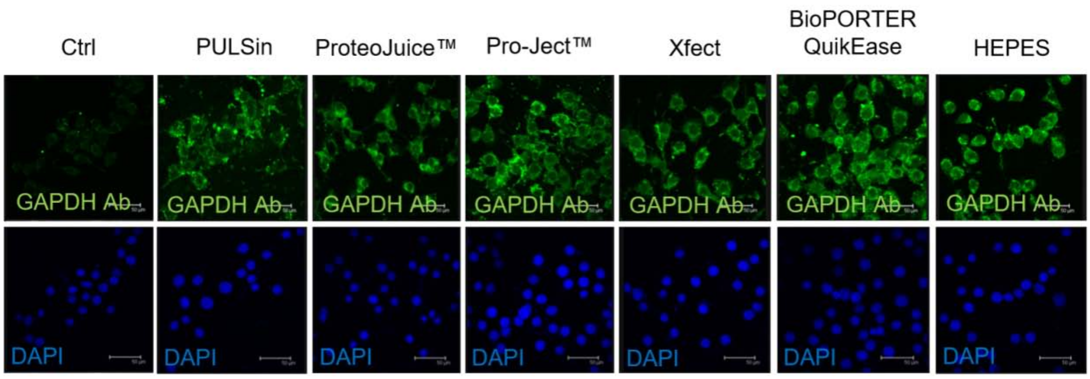

Figure S1. Anti-GAPDH antibodies were delivered into MDAH2774 cells using HEPES (20 mM), as described in the HEPES method, or using various commercially available protein transfection reagents (PULsin, ProteoJuice^{TM}, Pro-Ject^{TM}, Xfect, and BioPORTER QuickEase). After 24 h of incubation, cells were stained with Alexa Fluor 488–conjugated anti-mouse IgG antibodies and assayed using a confocal fluorescent microscope.
